# In regards to Chu et al.: Patterns of brain metastasis immediately before prophylactic cranial irradiation (PCI): implications for PCI optimization in limited-stage small cell lung cancer

**DOI:** 10.1186/s13014-020-01680-7

**Published:** 2020-11-02

**Authors:** Lukas Käsmann, Chukwuka Eze, Julian Taugner, Farkhad Manapov

**Affiliations:** 1 Department of Radiation Oncology, University Hospital LMU Munich, Munich, Germany; 2 German Center for Lung Research (DZL), Partner Site Munich, Munich, Germany; 3 German Cancer Consortium (DKTK), Munich, Germany

## Abstract

We read the article entitled “Patterns of brain metastasis immediately before prophylactic cranial irradiation (PCI): implications for PCI optimization in limited-stage small cell lung cancer” with great interest. In that study, the author reported about the importance of PCI timing in limited stage small cell lung cancer (LS-SCLC) in the era of MRI surveillance. In addition, the authors raise the issue of neurotoxicity of PCI. In this letter, we aimed to clarify the value of PCI in LS-SCLC and present ongoing trials regarding PCI and MRI surveillance in SCLC. As a result, we see the need for the development of a prediction tool to estimate the risk of intracranial relapse in LS-SCLC after chemoradiotherapy in order to support shared decision making through improved guidance.

Historically, brain as a metastatic site has a special importance in limited stage small cell lung cancer (LS-SCLC) [1, 2]. Earlier studies reported more than 50% cumulative risk of symptomatic brain metastases (BM) 2 years after initial diagnosis. Poor median survival after development of intracranial relapse was also documented. Prophylactic cranial irradiation (PCI) was shown to significantly reduce the incidence of symptomatic BM, especially, in the first 2 years after treatment. Based on the clinical practise guideline of ASTRO in 2020, PCI is strongly recommended for LS-SCLC (stage II or III) after response to chemoradiation [3]. Importantly, patients with higher risk of neurocognitive decline after PCI should be critically considered and treatment should be based on shared decision-making.

Conversely, the pivotal meta-analysis by Aupérin et al. could not demonstrate an effect of PCI on other metastases and local or regional recurrences [4]. Additionally, data on the occurrence of extracranial progression were only available for 67% of the cohort. Hence, no clear conclusion concerning application of PCI and extracranial disease control could be made [4].

Implementation of MRI surveillance has changed the significance of PCI in LS-SCLC. In 2008, the first retrospective study reported on the importance of a second contrast-enhanced cranial MRI immediately before the start of PCI for detection of occult intracranial relapse in LS-SCLC patients after chemoradiotherapy (CRT) [5]. In the same year, Seute et al. [6] described that implementation of cranial MRI at initial diagnosis of SCLC leads to a significant increase in the estimated prevalence of BM. Another important finding of the study was the prognostic role of a single intracranial metastasis in SCLC [6]. In 2015, a retrospective study conducted by Ozawa et al. at evaluated effects of cranial MRI and subsequent stereotactic radiation after completion of primary treatment in LS-SCLC and did not confirm a survival benefit of PCI [7]. However, the study was criticized for unbalanced treatment groups concerning disease stage and primary treatment. Another retrospective analysis from Mamesaya et al. [8] reported no progression-free and overall survival difference in the PCI versus non-PCI patients treated with CRT. Importantly, in both Japanese studies no significant difference in the cumulative incidence of BMs in the PCI and non-PCI subgroups was documented (Mamesaya et al.: 24.0% vs. 27%, *p* = 0.404; Ozawa et al.: 45.5 vs. 30.8% in a 2-year period, *p* = 0.313).

In the last years, numerous retrospective studies have confirmed the prognostic role of PCI after CRT [1]. Studies dedicated to the identification of risk factors potentially responsible for the development of intracranial relapse in LS-SCLC revealed TNM stage and tumor size as well as response to and duration of the CRT as highly relevant [1, 2]. Also timing of PCI after completion of CRT was shown to influence its efficacy [1]. Several studies have reported that “early” PCI is significantly more effective for control of subclinical BM than “late” PCI [4, 9]. Importantly, timing of PCI in most of these studies was associated with CRT duration. CRT duration itself was also reported to have a prognostic role in LS-SCLC [1].

Obviously, we need to develop a prediction tool estimating the risk of intracranial relapse in LS-SCLC after CRT. This tool may be based on the previously described tumor- and treatment-derived factors, including TNM stage, tumor size, CRT duration and timing of PCI and define patients which could maximally benefit from application of PCI. The prognostic role of the number of BMs (single vs. multiple) and concomitant symptomatic burden must also be evaluated. Additionally, the individual risk of acute and sub-acute toxicity should be identified and factored into shared decision-making.

Finally, the development of a prediction tool of BM could be performed in the ongoing prospective studies which incorporate a comprehensive MRI surveillance in the treatment of LS-SCLC (see Table 1). The results of these studies will define the true role of PCI in LS-SCLC and may help to achieve adequate patient selection.

**Table 1:** Prospective trials regarding MRI surveillance and PCI in small cell lung cancer (SCLC)

Name of the study	Phase	Status
MRI brain surveillance alone versus MRI surveillance and prophylactic cranial irradiation (PCI): a randomized Phase III trial in small-cell lung cancer (Maverick)	Phase-3	Open, recruiting
Watchful observation of patients with limited small cell lung cancer instead of the PCI—prospective, multicenter one-arm study (PCILESS)	Phase-2	Open, recruiting

## Data Availability

No data generated.

## Abbreviations

ASTRO: American Society for Radiation Oncology
BM: Brain metastasis
CRT: Chemoradiotherapy
LS-SCLC: Limited stage small cell lung cancer
MRI: Magnetic resonance imaging
PCI: Prophylactic cranial irradiation
SCLC: Small cell lung cancer
TNM: Tumor node metastasis
